# Expression of Beta-Catenin, Cadherins and P-Runx2 in Fibro-Osseous Lesions of the Jaw: Tissue Microarray Study

**DOI:** 10.3390/biom12040587

**Published:** 2022-04-16

**Authors:** Giuseppe Pannone, Riccardo Nocini, Angela Santoro, Francesca Spirito, Pier Francesco Nocini, Silvana Papagerakis, Renny T. Franceschi, Marina Di Domenico, Angelina Di Carlo, Nana Danelia, Lorenzo Lo Muzio

**Affiliations:** 1 Department of Clinical and Experimental Medicine, University of Foggia, Viale Pinto 1, 71122 Foggia, Italy; giuseppe.pannone@unifg.it (G.P.); lorenzo.lomuzio@unifg.it (L.L.M.); 2 Section of Ear Nose and Throat (ENT), Department of Surgical Sciences, Dentistry, Gynecology and Pediatric, University of Verona, 37126 Verona, Italy; riccardo.nocini@gmail.com; 3 Operative Unit of Gynecological and Breast Pathology, Department of Sciences of the Women and Child Health, Fondazione Policlinico Agostino Gemelli—UCSC, 00168 Rome, Italy; angelasantoro1@hotmail.it; 4 Department of Oral and Maxillofacial Surgery, University of Verona, 37126 Verona, Italy; pierfrancesco.nocini@univr.it; 5 Department of Surgery, College of Medicine, University of Saskatchewan, 107 Wiggins Road, Saskatoon, SK S7N 5E5, Canada; silvana.papagerakis@usask.ca; 6 Department of Periodontics and Oral Medicine, School of Dentistry, University of Michigan, Ann Arbor, MI 48109, USA; rennyf@umich.edu; 7 Department of Precision Medicine, University of Campania, 80138 Naples, Italy; marina.didomenico@unicampania.it; 8 Department of Medico Surgical Sciences and Biotechnologies, “Sapienza” University of Rome, 00185 Rome, Italy; angelina.dicarlo@uniroma1.it; 9 Albius Dental Center, 27 Revaz Tabukashvili st., 0108 Tbilisi, Georgia; nanadanelia71@gmail.com; 10 C.I.N.B.O. (Consorzio Interuniversitario Nazionale per la Bio-Oncologia), 66100 Chieti, Italy

**Keywords:** β-catenin, bone disease, cadherin, fibro-osseous lesions, fibrous dysplasia, HPT-JT syndrome, McCune–Albright, P-Runx2, tissue microarray, TMA

## Abstract

Fibrous dysplasia (FD) and hyperparathyroidism-jaw tumor syndrome (HPT-JT) are well-characterized benign bone fibro-osseous lesions. The intracellular mechanism leading to excessive deposition of fibrous tissue and alteration of differentiation processes leading to osteomalacia have not yet been fully clarified. Tissue Microarray (TMA)-based immunohistochemical expression of β-catenin, CK-AE1/AE3, Ki-67, cadherins and P-Runx2 were analyzed in archival samples from nine patients affected by FD and HPT-JT and in seven controls, with the aim of elucidating the contribution of these molecules (β-catenin, cadherins and P-Runx2) in the osteoblast differentiation pathway. β-catenin was strongly upregulated in FD, showing a hyper-cellulated pattern, while it was faintly expressed in bone tumors associated with HPT-JT. Furthermore, the loss of expression of OB-cadherin in osteoblast lineage in FD was accompanied by N-cadherin and P-cadherin upregulation (*p* < 0.05), while E-cadherin showed a minor role in these pathological processes. P-Runx2 showed over-expression in six out of eight cases of FD and stained moderately positive in the rimming lining osteoblasts in HPT-JT syndrome. β-catenin plays a central role in fibrous tissue proliferation and accompanies the lack of differentiation of osteoblast precursors in mature osteoblasts in FD. The study showed that the combined evaluation of the histological characteristics and the histochemical and immunohistochemical profile of key molecules involved in osteoblast differentiation are useful in the diagnosis, classification and therapeutic management of fibrous-osseous lesions.

## 1. Introduction

Benign fibro-osseous lesions (BFOLs) are a clinically and pathogenically different group of bone disorders that share similar histologic aspects, and they are characterized by mesenchymal hyperproliferation in the form of hyper-cellular fibrous tissue formation alternating with areas of ossification. In fact, a common finding is the replacement of normal bone with a fibrous tissue containing varying amounts of mineralized substance. BFOLs include developmental pathologies, reactive or dysplastic processes and neoplasms [1,2]. Among BFOLs, two diseases have been well-characterized, fibrous dysplasia (FD) and hyperparathyroidism-jaw tumor syndrome (HPT-JT) (OMIM:145001), caused by specific gene mutations, GNAS and HRPT2/CDC73, respectively [3,4]. However, the exact molecular mechanisms by which the gene mutations transfer pathological signals in bone precursor cells and differentiated cells is not well understood. 

FD is a benign dysplastic process of altered osteogenesis that may occur in a single bone (monostotic) or in multiple bones (polyostotic) [5]. The presence of the classic triad (bone abnormalities/polyostotic FD, patchy skin pigmentation and endocrine disorders such as early puberty) allows the diagnosis of a rare condition, called McCune–Albright syndrome (OMIM:174800) [6]. It has an estimated prevalence between 1/100,000 and 1/1,000,000 [7].

It is difficult to define the true FD incidence and prevalence; it has been reported to represent approximately nearly 1% of primary bone tumors, accounting for approximately 7% of benign bone tumors with the monostotic form reported to be six to ten times more common than the polyostotic form. It is considered a disorder of growing bones, so the first lesions are identified in adolescents [8].

FD has been associated with a mutation in the gene GNAS1 that encodes the G protein alpha subunit (Gs-alpha) coupling cAMP to hormone receptors [3,9]. Monostotic fibrous dysplasia occurs in the jaws, frontal, ethmoidal, temporal and calvarial bones [10]. Clinically, the maxilla is affected more often than the mandible, and diagnosis is made frequently during the first and the second decade of life. The disease is characterized by painless osseous expansion with facial asymmetry. Radiographic features are variable depending upon the stage of the disease, but typically characterized by a homogeneous diffuse radiopacity with a “ground glass” appearance of the border area which becomes more sclerotic as the disease progresses [11]. The microscopic picture consists of narrow and curved trabeculae depicting a woven bone with a fish-hook aspect, even if there is an important overlapping of histopathological features with other fibro-osseous lesions such as ossifying fibroma and osseous dysplasia. Therefore, the simultaneous analysis of the clinical and radiological characteristics, along with microscopic aspects, is essential for the diagnosis [12].

Osteogenesis is arrested at an early stage without achieving terminal differentiation with an osteomalacic osteoid appearance consisting of anastomosing trabeculae that can be lined by abnormal fibroblast-like osteoblasts. Some cases show woven trabeculae with the classic “Chinese figures” or a mosaic pattern of resting and reversal lines [12]. 

Hyperparathyroidism-jaw tumor syndrome (HPT-JT) is an early onset and highly penetrant autosomal dominant disease due to mutation of the HRPT2/CDC73 gene located on 1q25-31 [13]. HRPT2/CDC73 is a gene encoding for parafibromin [4], a nuclear protein with a well-defined tumor suppressor function, lost in HPT-JT-associated tumors [14]. The exact prevalence of hyperparathyroidism-jaw tumor syndrome is unknown. HPT-JT syndrome has also been associated with other benign and malignant diseases, such as renal, uterine cervix, testis, colon and pancreatic tumors. Association between jaw lesions and familial hyperparathyroidism was firstly described by Kennet and Pollick in the early 1970s [15]. About 50% of affected patients develop fibro-osseous tumors of the maxilla or mandible, which may recur, are independent of the course of the parathyroid adenomas and are distinct from brown tumors associated with sporadic severe hyperparathyroidism [16]. 

The differential diagnosis between the various types of fibro-osseous lesions is often difficult due to significant overlaps of the clinical, radiological and histopathological characteristics. An integration of all aspects is therefore essential. Furthermore, currently several immunohistochemical biomarkers are being studied as auxiliary factors for diagnosis [12,17].

The osteoblastic differentiation is divided in different stages, including proliferation, maturation and terminal differentiation [18]. Numerous molecules and different pathways are involved in the osteoblastic differentiation process, chondrocyte maturation, bone formation and remodeling, including Hedgehogs, TGF-beta, BMPs, PTH and WNTs [19].

In the diagnosis of fibrous dysplasia, a potential biomarker could be the detection of the mutated GNAS1 gene; however, it is a variable parameter due to the coexistence of mutated and wild-type GNAS1 within the lesion, as a result of a genetic mosaicism; it is also a gene that appears to be mutated in different exons in other pathologies [12].

Instead, a role could be played by the study of the Wnt/b-catenin pathway, which appears to be upregulated due to Gs-alpha activating mutations. Recent studies have shown how the detection of positive nuclear β-catenin can allow to exclude fibrous dysplasia during differential diagnosis between the various fibro-osseous lesions. This is because from immunohistochemical investigations it emerged that almost all the fibro-osseous lesions showed β-catenin nuclear positivity, while most of the fibrous dysplasias were negative for nuclear staining [17].

Cadherins are a group of ubiquitous transmembrane glycoproteins responsible for adhesion between heterotypic cells and for maintaining the correct tissue architecture. The cadherin family can be divided into classical cadherins, unconventional cadherins, desmosomials and protocadherins [20].

Classical cadherins are epithelial cadherin (E-cadherin), placental cadherin (P-cadherin) and neuronal cadherin (N-cadherin) and represent a class of adhesion molecules that interact with catenins through the cytoplasmic domain. They play a fundamental role in tissue homeostasis, tissue morphogenesis, cell differentiation and carcinogenesis [21]. Their expression is linked to that of the catenins, so any deregulation of the cadherin–catenin complex can result in an altered tissue development and tumorigenesis [22].

In this work, in order to elucidate the contribution of different molecules (β-catenin, cadherins and P-Runx2) in the osteoblast differentiation pathway, we analyzed their specific immunohistochemical expression profiles in the mesenchymal component (osteoblasts and fibroblasts) of two types of fibro-osseous lesions, in different types of reactive fibrous tissue and in the remodeling bone, in order to highlight, along with the analysis of their correlation with histological patterns, their possible role as diagnostic auxiliaries.

## 2. Materials and Methods

### 2.1. Patients and Histological Classification of Samples

Archival formalin blocks of 9 patients affected by fibrous lesions of the jaw were retrieved from the archive of University of Foggia and Verona, Italy (Table 1) from January 2018 to December 2021. Criteria of inclusion were: the histological diagnosis of fibro-osseous lesion of the jaws, re-analyzed according to the Eversole LR et al. classification [23]. Cases fulfilling the diagnosis of FD were further classified according to the histopathological pattern of Riminucci M et al. [24,25]. In brief, Riminucci sub-divides the FDs in three categories: 1, pagetoid; 2, ‘Chinese writing’; 3, hyper-cellular [24,25]. Sclerotic-pagetoid pattern appears with bone trabeculae fully connected together; the Chinese alphabet pattern is characterized by thin bone areas, sometimes curved, fully conjugated. The sclerotic hyper-cellulated pattern, instead, contains an abundant amount of immature bone tissues, constituted by osseous discontinuous trabeculae, distributed in an ordered, often parallel pattern [25]. Criteria of exclusion: lesions with overlapping features between two distinct diagnostic entities, or in which consensus could not be reached between the authors, were excluded; odontogenic tumors, low-grade osteosarcoma, osteomyelitis and aneurysmal bone cyst were excluded as well. The selected cases were: one patient was affected by hyperparathyroidism-jaw tumor syndrome (HPT-JT), caused by specific HRPT2/CDC73 gene mutations. Monostotic, polyostotic FD and McCune–Albright-Sternberg syndrome was confirmed in a group of 8 patients by endocrine system examinations with dosage of hormones, by ultrasound scans, X-rays of the skeleton, skull CT scan, dermatological detection of café-au-lait pigmentations and later confirmed by the molecular study of the GNAS1 gene. The subjects had undergone surgery for jaw deformity in the years 1982–1990, and therefore had not been subjected to medical therapy with bisphosphonates because it was not yet studied and approved in those years for this purpose. Surgical samples were taken during operations intended to correct facial deformity. Finally, in the study we also included 7 controls. Criteria of inclusion were: reactive fibromyxoid tissue, reactive mature fibrous tissue, normal bone with hematopoietic cells and remodeling bone surrounding developmental odontogenic cyst.

All patients gave their written informed consent, and the study was approved by the Ethics Committee of the Azienda Ospedaliero-Universitaria of Foggia.

### 2.2. Microscopic and Histochemical Evaluations

**Figure 1:**
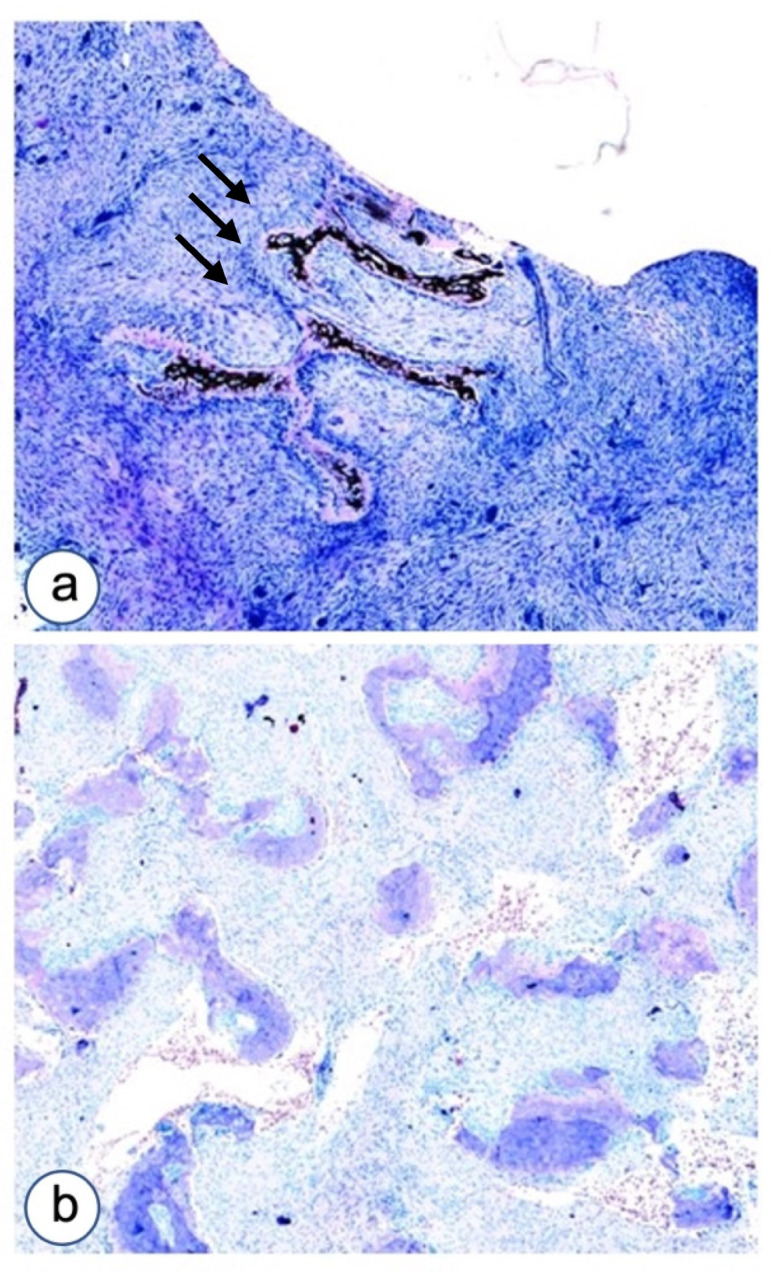
Microscopic aspects of impaired differentiation in Fibrous dysplasia of the jaw as evaluated by Von Kossa–Giemsa histochemical staining. (**a**) A case with Chinese ideogram aspect and high cellularity composed by fibroblastoid cells committed to osteoblast differentiation associated with giant cells. (**b**) Another case of FD with curved thin trabeculae of osteoid with high cellularity. Magnifications, ×4 (**a**,**b**).

Serial 4-micron section was cut and stained with hematoxylin and eosin, Van Gieson and Von Kossa counterstained with Giemsa, in order to classify the histopathological pattern of the lesions and to value the proportions of osteoid versus calcified bone (Figure 1).

The following histological characteristics with corresponding scores were evaluated:

- Histologic pattern: (1), sclerotic-pagetoid; (2), Chinese alphabet; (3), sclerotic hyper-cellulated.
- Cellularity: (1), low; (2), moderate; (3), high.
- Hemorrhage: (0), negative; (1), positive.
- Osteoids rimmed by fibroblast-like osteoblasts: (0), negative; (1), focal; (2), moderate; (3), high.

### 2.3. Tissue Microarray (TMA) Construction

Tissue cores of 3 mm in diameter were transferred from the donor paraffin inclusion blocks in a receiver block to set up a Tissue Microarray (TMA) according to the already described method [26].

### 2.4. TMA-Based Immunohistochemistry

**Figure 2:**
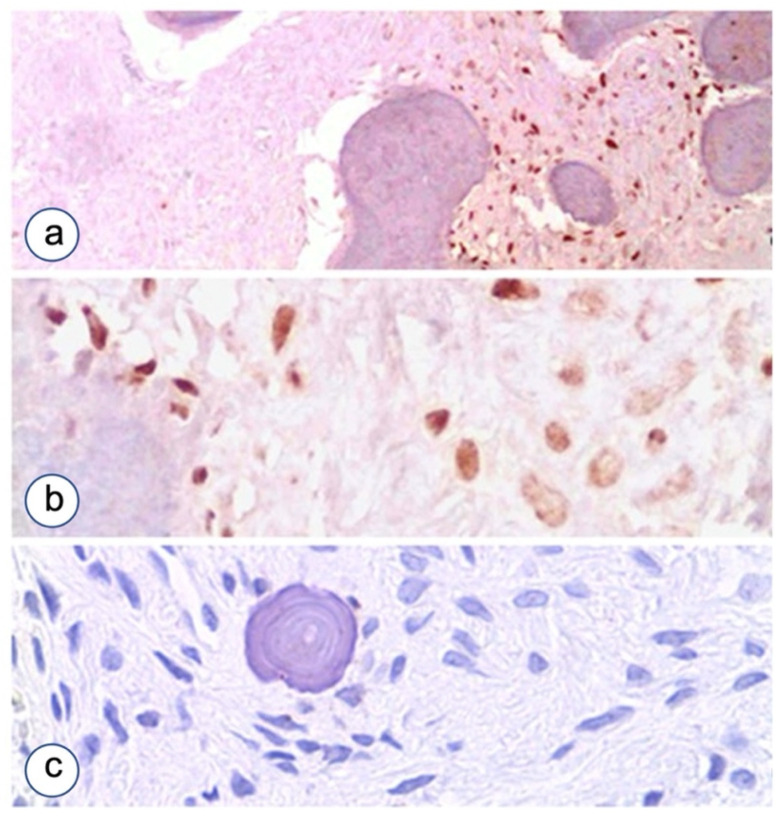
Cytokeratin cocktail in HPT-JT syndrome and fibrous dysplasia. CKAE1/AE3 is expressed in maxillary tumor associated with HPT-JT syndrome. (**a**), Further magnification in (**b**), but it stains negative in FD even in cases with high cellularity. (**c**) LSAB-HRP, nuclear counterstaining with hematoxylin. Magnifications, ×10 (**a**), ×40 (**b**,**c**).

Four-microgram serial sections from formalin fixed and paraffin embedded TMA blocks were cut and mounted on poly-L-lysine-coated glass slides. Immunostaining was performed by linked streptavidin-biotin horseradish peroxidase technique (LSAB-HRP). After sequential deparaffinization and hydration, the slides were treated with 0.3% H_2_O_2_ for 15 min to quench endogenous peroxidase. Antigen retrieval was performed by microwave heating—a 1st time for 3 min at 650 W, a 2nd and a 3rd time for 3 min at 350 W—and the slides immersed in 10 mM citrate buffer pH 6. After heating, the sections were blocked for 60 min with 1.5% horse serum (Santa Cruz Biotechnology, Dallas, TX, USA) diluted in PBS buffer before reaction with the primary antibody. Primary Ab was diluted with 0.05 M Tris-HCl buffer pH 7.4 containing 1% bovine serum albumin and incubated at optimal dilution and time. The primary Abs and conditions for their use were: 1:100—diluted mouse monoclonal anti-cytokeratin (Pan AE1/AE, Ventana, Tucson, AZ, USA; 1:250) (Figure 2); rabbit monoclonal β-catenin mAb (6B3, Cell Signaling Technology, Danvers, MA, USA); 1:100, monoclonal mouse antibody (MIB 1, DAKO, Glostrup, Denmark); 1:500, rabbit polyclonal anti human N-cadherin Ab (N-cadherin N-19, SC-1502—Santa Cruz Biotechnology, Dallas, TX, USA); 1:250, monoclonal anti-E-cadherin Ab (clone 36, Transduction Laboratories, Lexington, KY, USA); 1:25, rabbit polyclonal anti-CDH11 Ab (code GTX109792, GeneTex, Irvine, CA, USA); 1:250, monoclonal anti P-cadherin (BD Transduction Laboratories, Franklin Lakes, NJ, USA); 1:500, anti P-Runx2 (kindly provided by prof. Renny Franceschi (University of Michigan, Ann Arbor, MI, USA), a rabbit polyclonal antibody specifically detecting Runx2-S319-Phosphorylation [27].

The accuracy and the specificity of these antibodies have been previously tested in our different scientific works [28,29,30,31,32,33]. After two washes with PBS, the slides were treated with biotinylated species-specific secondary antibodies and streptavidin-biotin enzyme reagent (DAKO, Glostrup, Denmark), and the color developed by 3,3′-diaminobenzidine tetrahydrochloride. Sections were counterstained with Mayer’s hematoxylin and mounted using xylene-based mounting medium. Negative control slides without primary antibody were included for each staining. The results of the immunohistochemical staining were evaluated separately by two observers. Two of the Authors recorded, blindly and independently, the same slides of each case to evaluate the inter-observer variation by the K test. Immunostained cells were counted in each spot in at least 4 high power fields (HPFs) analyzed by a light microscope (Olympus, BX53, Olympus Corporation, Tokyo, Japan). For each case, the mean percentage of positive cells in all sections examined was determined and scored as follows: 0, negative; 1+, <25%; 2+, 25–50%; 3+, 50–75%; 4+, >75%. The sub-cellular distribution of β-catenin was evaluated as follows: M, membranous; C, cytoplasmic; C-M, mainly cytoplasmic with focal membranous staining; C-N, mixed cytoplasmic and nuclear staining, mainly cytoplasmic.

### 2.5. Statistical Analysis

The data were analyzed by the Stanton Glantz statistical software 3 (MS-DOS) and GraphPad Prism software version 8.00 for Windows (Graph Pad software, San Diego, CA, USA; www.graphpad.com, access date: 21 September 2021). Differences between groups were determined using the one-way analysis of variance (ANOVA) and the Student–Newman–Keuls test. Only *p* values < 0.05 were considered significant.

## 3. Results

### 3.1. Histopathological Analysis

#### 3.1.1. Fibrous Dysplasia

Microscopic findings are summarized in Table 2. The main histological pattern of FD in the jaws were the pagetoid type and the Chinese alphabet. The sclerotic-hyper-cellular type was expressed in only one case, in association with the pagetoid pattern. High/moderate cellularity was reported in five out of eight cases of FD. Focal/moderate presence of rimming osteoblasts was observed in all studied FD cases.

#### 3.1.2. HPT-JT Syndrome

In the single case of HPT-JT syndrome, we observed pagetoid morphology of malacic non-mineralized bone, scanty cellularity and osteoblastic differentiation of cells appearing as the lining type.

#### 3.1.3. Controls

In the reactive processes, no bone formation was observed; in remodeling bone, parallel osseous trabeculae with focal fish-hook appearance were noted. Generally, prevalent moderate cellularity was reported, with the focal/moderate presence of rimming osteoblasts in three control cases.

### 3.2. Immunohistochemistry

TMA-based immunohistochemical findings in fibro-osseous lesions of the jaws are synthetically reported in Table 2**.**

#### 3.2.1. Fibrous Dysplasia

**Figure 3:**
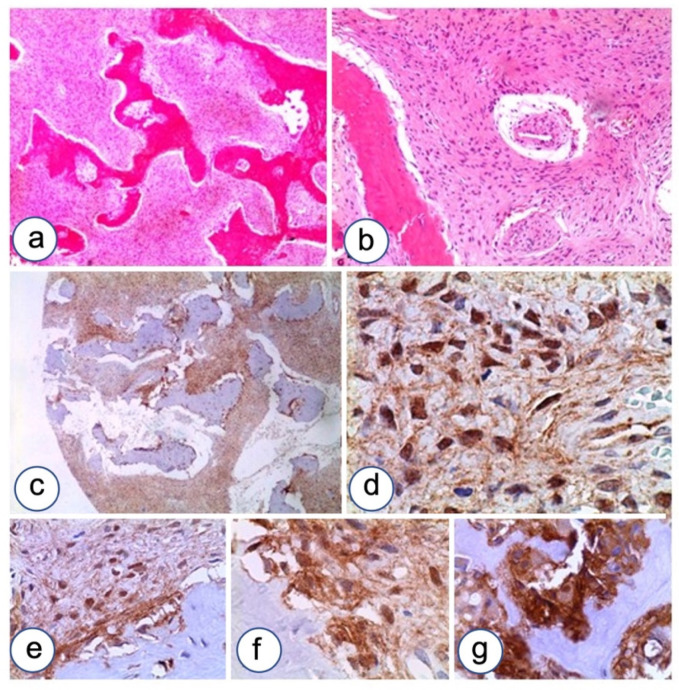
B-catenin over-expression in FD. (**a**,**b**) A representative case of FD with Chinese ideogram pattern (hematoxylin and eosin) showing strong immunostaining for β-catenin (**c**), with cytoplasmic-nuclear delocalization (**d**), especially localized in the area surrounding dysplastic bone trabeculae lined by retracted osteoblasts (**e**–**g**); LSAB-HRP, nuclear counterstaining with Gill’s type II hematoxylin. Magnifications, ×4 (**a**,**c**), ×10 (**b**,**e**), ×20 (**f**,**g**), ×40 (**d**).

**Figure 4:**
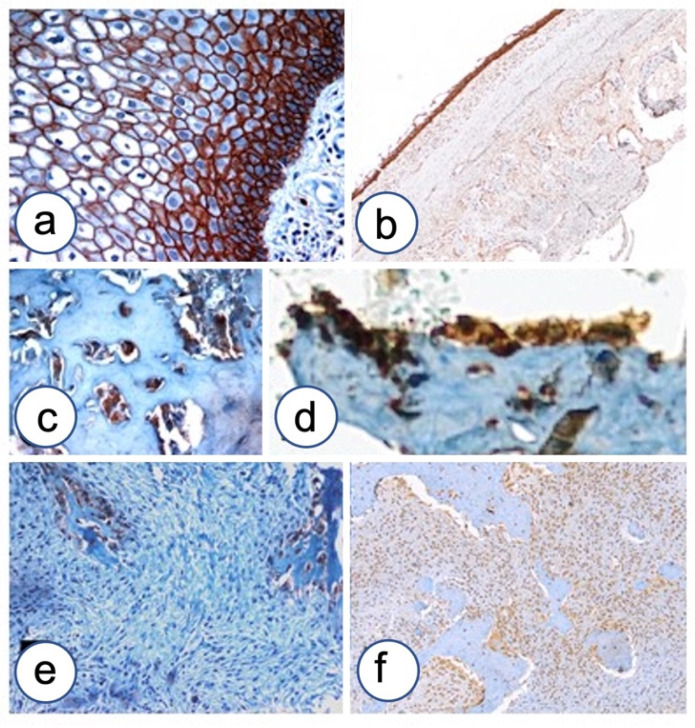
N-cadherin upregulation in FD. (**a**) Positive control for N-cadherin staining in oral epithelium. (**b**) N-cadherin staining in epithelium of developmental odontogenic cyst and surrounding remodeling bone. (**c**) N-cadherin over-expression in osteoblasts lining osteoid in a case of pagetoid-type FD. (**d**) Magnification of osteoblasts expressing N-cadherin in FD. (**e**) A case of FD showing N-cadherin selectively expressed in osteoblasts without any expression in fibroblastoid cells. (**f**) A case of FD showing N-cadherin over-expression in fibroblastoid cells committed to inefficient osteoblast differentiation. Magnifications, ×4 (**b**,**c**), ×10 (**e**,**f**), ×20 (**a**,**d**).

β-catenin expression was detected in five out of eight FD cases of the jaw (Figure 3). Cytoplasmic localization was observed in all positive cases, two of which revealed a mixed staining with focal membranous and nuclear expression of the study protein. N-cadherin showed constant and significant upregulation in FD compared to normal controls (*p* < 0.05) (Figure 4). N-cadherin over-expression was associated with high cellularity and low percentage of bone and osteoid formation. Over-expression was detected in osteoblasts lining the osteoid. FD showed a selective N-cadherin expression in osteoblasts. Differently, in another case N-cadherin over-expression was observed in fibroblastoid cells committed to inefficient osteoblast differentiation.

**Figure 5:**
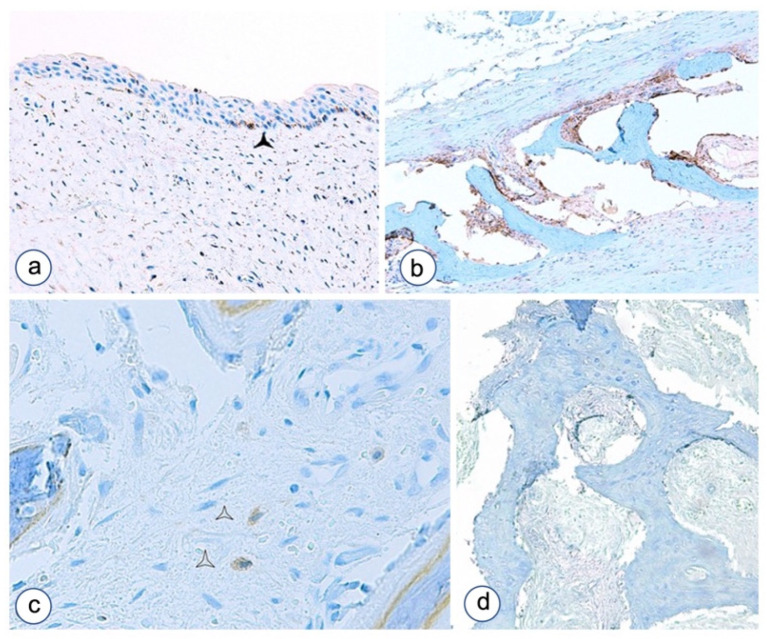
OB-cadherin lack of expression in FD of the jaws. (**a**) Positive control of OB-cadherin in basal layer of developmental odontogenic cyst (black star) and focal fibroblasts of connective tissue wall. (**b**) Strong expression of OB-cadherin in the remodeling bone surrounding odontogenic cyst, further magnification of Figure 3c. (**c**) Precursors of osteoblasts OB-cadherin positive in mesenchymal stroma during normal bone remodeling (empty stars). (**d**) OB-cadherin lack of expression in FD of the jaw; LSAB-HRP, nuclear counterstaining with Gill’s type II hematoxylin. Magnifications, ×4 (**b**,**d**), ×10 (**a**), ×20 (**c**).

**Figure 6:**
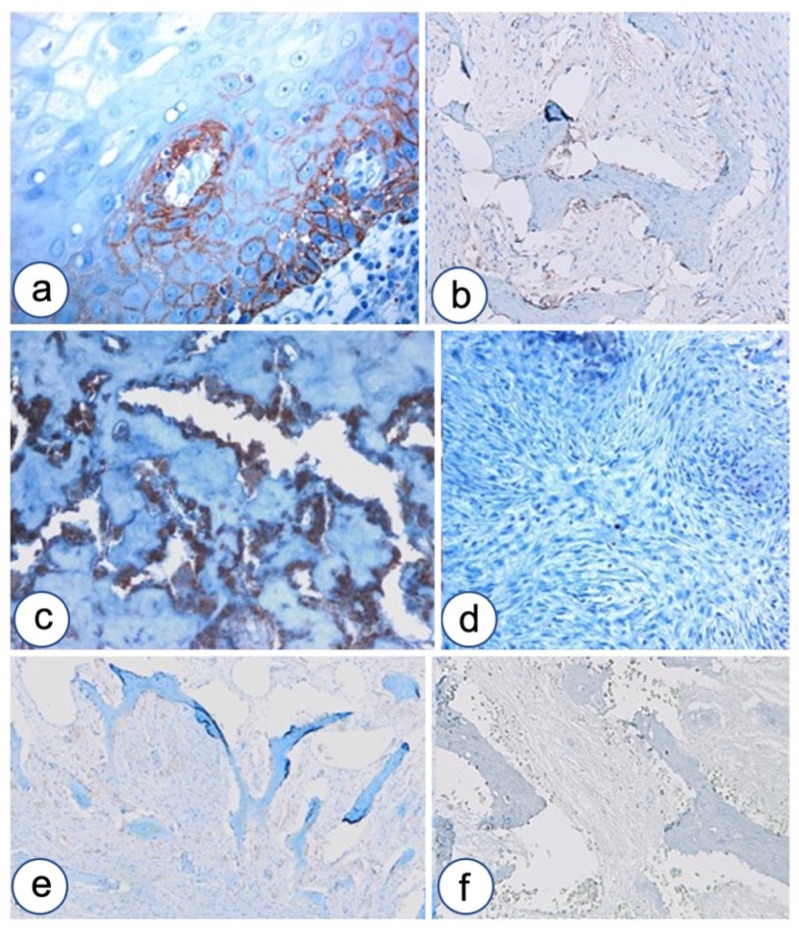
P-cadherin and E-cadherin expression in FD of the jaw. (**a**) P-cadherin expression in oral epithelium. (**b**) P-cadherin expression in remodeling bone surrounding developmental odontogenic cyst. (**c**) P-cadherin over-expression in pagetoid FD is confined to osteoblasts. (**d**) P-cadherin negative staining in fibrous area of FD without osteoid formation. (**e**) E-cadherin shows faint staining in remodeling normal bone. (**f**) E-cadherin negative staining in FD of the jaw. Magnifications, ×4 (**e**,**f**), ×10 (**b**,**c**), ×20 (**a**,**d**).

**Figure 7:**
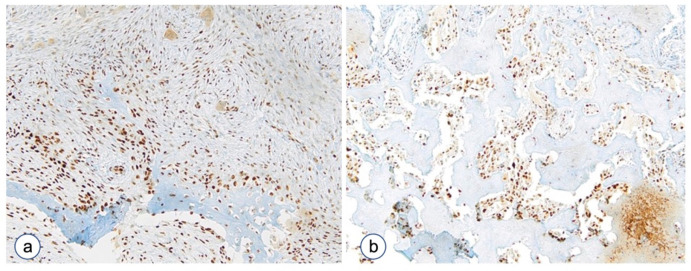
P-Runx2 staining in FD of the jaw. P-Runx2 over-expression in representative cases of FD. (**a**) Thin trabeculae type. (**b**) Pagetoid type. Magnifications, ×4 (**b**), ×10 (**a**).

OB-cadherin stained medullar precursors committed toward osteoblast differentiation and terminally differentiated osteoblasts in bone controls, while it stained negative in seven out of eight cases of FD analyzed. It was weakly expressed only in one case with the Chinese alphabet pattern (Figure 5). P-cadherin showed over-expression in pagetoid/hyper-cellulated FD, confined to osteoblasts, while a negative staining was observed in the fibrous area of FD without osteoid formation (Figure 6). Weak expression was also detected in a case of FD with the Chinese alphabet pattern. The E-cadherin antibody stained negative in all pathological cases examined, and the comparison with normal bone controls resulted statistically significant (*p* < 0.05) (Figure 6). Finally, P-Runx2 also showed over-expression in six out of eight cases of FD, both in fibroblastic stroma and in retracted osteoblasts (Figure 7). 

#### 3.2.2. HPT-JT Syndrome

β-catenin and N-cadherin demonstrated faint immunostaining; E-cadherin, P-cadherin and OB-cadherin resulted negative. P-Runx2 stained positive in the rimming lining osteoblasts.

#### 3.2.3. Controls

**Figure 8:**
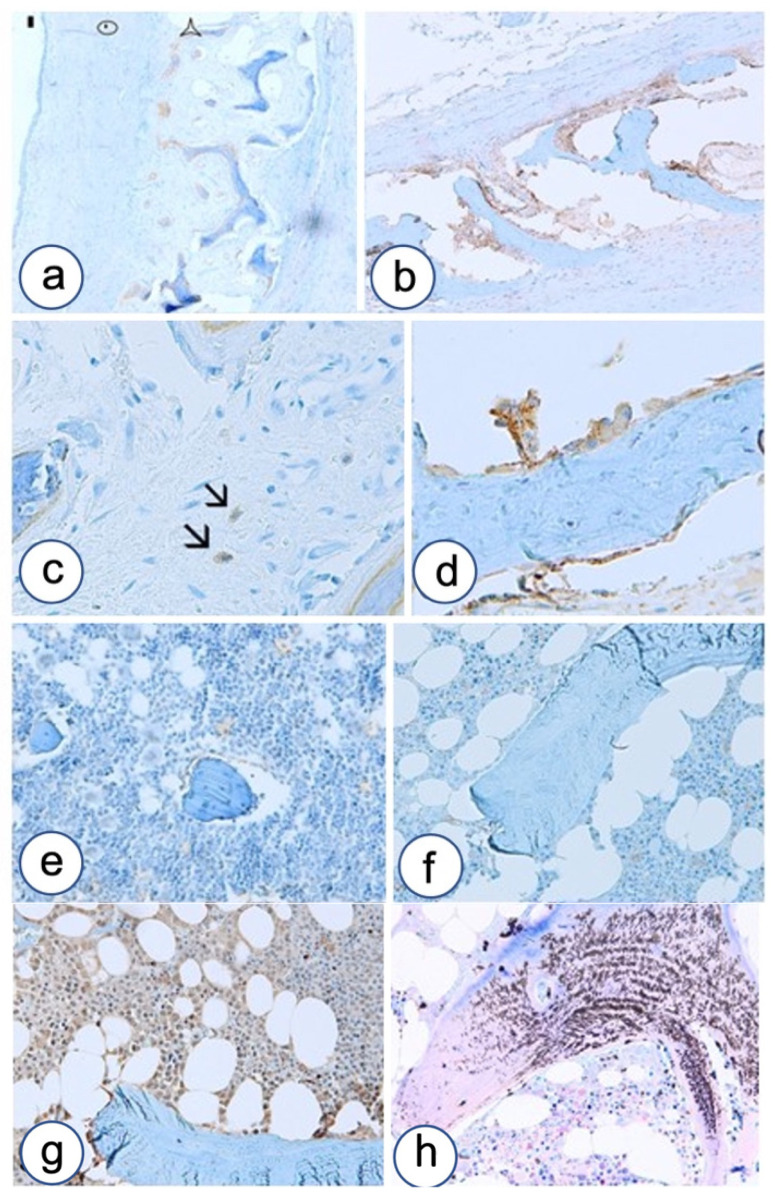
B-catenin, OB-cadherin, E-cadherin, N-cadherin and bone mineralization in controls of non-dysplastic bone as evaluated by immunohistochemistry and Von Kossa–Giemsa staining. (**a**) Overview showing odontogenic cyst composed of epithelial layer (rectangle), and connective wall (circle) compressing and remolding normal jaw bone (star), the latter showing OB-cadherin staining surrounding bone trabeculae. (**b**) Further details of OB-cadherin positive bone. (**c**) Precursors of OB-cadherin positive osteoblasts (arrows). (**d**) Details of OB-cadherin positive osteoblasts. (**e**) B-catenin staining in normal osteomedullar biopsy showing faint expression in medullar precursors and osteoblasts. (**f**) E-cadherin staining in normal osteomedullar biopsy showing faint expression in medullar precursors and negative staining of osteoblasts. (**g**) N-cadherin staining in normal osteomedullar biopsy showing moderate expression in medullar precursors and osteoblasts. (**h**) Control of normal bone mineralization and medullar precursors by Von Kossa–Giemsa staining. Magnifications, ×4 (**a**), ×10 (**b**,**c**,**e**,**g**,**h**), ×20 (**d**).

β-catenin showed faint membranous positive staining in three cases; E-cadherin was also weakly positive in three cases; N-cadherin revealed moderate intensity of the staining in three cases; OB-cadherin and P-cadherin were expressed only in bone tissue; P-Runx2 stained generally weakly positive in all the control cases (Figure 8).

#### 3.2.4. The Proliferative Index

The proliferative index was proven as rather inconsistent, except in some FD cases characterized by high cellularity in which ki-67 staining showed focal staining (data not shown). Altogether, FDs demonstrated that there are not cell cycle disorders and that the occurrence of a high amount of fibrous tissue is due to disorders of differentiation and apoptosis induction.

## 4. Discussion

The differential diagnosis of jaw FD and HPT-JT should be made comparing findings of benign fibro-osseous lesions of periodontal origin [34], osteofibrous dysplasia (ossifying fibroma), central giant cell reparative granuloma (cGCRG) [35], giant cell tumor [36], osteitis fibrosa cystic resulting from hyperparathyroidism, desmoplastic fibroma, aneurismal bone cyst, and in cases showing hyper-cellular pattern with low-grade aggressive mesenchymal tumors such as desmoid-type fibromatosis, aggressive fibromatosis and low-grade fibrosarcoma [37].

Interestingly, FD shares with aggressive fibromatosis the activation of the WNT-β-catenin pathway [31], which in turn activates cell cycle progression and tissue invasion at the expense of terminal differentiation. However, in aggressive fibromatosis the CTNNB1 gene encoding the β-catenin protein is frequently mutated [38]. On the contrary, in FD this gene is infrequently mutated, as recently shown [17]. Indeed, other routes to β-catenin over-expression and nuclear accumulation, different from mutation, are shown in the current literature. Non-mutational β-catenin accumulation may be mediated by different indirect mechanisms, for instance PDGF receptor activation [39], plakoglobin loss [40], epigenetic WNT inhibitor inactivation [41], parathyroid hormone action [42], and by constitutive activation of Gα proteins exerting their effects in modulating the WNT/β-catenin pathway competing for axin, therefore acting on the axin-containing β-catenin destruction complex, as recently showed by Regard JB in FD [43]. WNT/β-catenin signals control the osteoblast differentiation process pathway at least in three points. In particular, in the early stages β-catenin prevents stem cells from differentiating in osteo-chondroblast precursors [44], while in the more advanced stages β-catenin exerts stimulatory roles in differentiation of immature to mature osteoblasts and in terminal differentiation of osteocytes, maintaining bone homeostasis and preventing bone loss [18]. Moreover, a recent paper from our group demonstrated that catenins were expressed in cells with morphological characteristics of osteoblasts, especially in the areas of new bone formation at the junction between mineralized and unmineralized tissue, showing an overall involvement of catenins in human bone tissues and in particular during the bone regeneration process [29].

A β-catenin binding site located at the COOH terminus is the most conserved segment among an important family of transmembrane proteins involved in intercellular adhesion: the cadherins. Literature data have highlighted the important crosstalk between cell adhesion and WNT signaling molecules that impacts osteoblast function, bone formation and bone mass [39]. Classical cadherins are calcium-dependent hemophilic adhesion receptors, able to significantly influence the process of tissue differentiation [45]. During tissue development, the pattern of cadherins expressed in undifferentiated mesenchymal cells undergoes a number of changes until their transition into mature cell phenotypes [46]. Among the cadherin family members, four proteins are particularly important in the biology, physiology and pathology of the bone, and differently involved in the cell sorting, alignment and separation through differentiation of osteoblasts from pluripotent mesenchymal stem cells [47]: E-cadherin, N-cadherin, P-cadherin and OB-cadherin. In particular, E-cadherin, a type I cadherin, has been associated with bone invasion by cancer metastases [48], and N-cadherin and OB-cadherin with bone differentiation process [49]. Eric Haÿ et al. [50] studied the role of E-cadherin, together with N-cadherin, in the promotion of osteoblast differentiation and osteogenesis by BMP-2 in immortalized human neonatal calvaria (IHNC) cells. According to other studies, N-cadherin, a type I cadherin, interacts with axin and the WNT co-receptor LRP5, regulating canonical Wnt/β-catenin signaling in osteoblasts. This causes increased β-catenin ubiquitination and altered TCF/LEF transcription in response to the WNT signal, resulting in cell-autonomous defective osteoblast function, decreased osteoblast gene expression and osteogenesis, reduced bone formation and delayed bone mass acquisition [51]. P-cadherin, belonging to the type I cadherin family, although expressed in osteoblasts seems to have a minor role in intercellular adhesion and in osteogenesis [47]. OB-cadherin, also known as cadherin-11, a type II cadherin, was first identified in mouse osteoblasts. It is normally expressed in cells with a mesenchymal phenotype, and in the kidney and brain during development [52]. In particular, OB-cadherin is expressed preferentially in osteoblasts, with only weak signals detectable in brain, lung and testicular tissues [53]. OB-cadherin, like other classical cadherins, is composed of an extracellular domain with five repeated sub-domains (EC 1–5), a single transmembrane domain and a cytoplasmic C-terminal tail. The calcium binding sites are located in the extracellular domain and participate in the homodimerization of cadherin present on neighboring cells [54]. Expression of OB-cadherin is associated with osteoblast differentiation and has been proposed to function in cell sorting, migration and alignment during the maturation of osteoblasts [47]. As a result, OB-cadherin has also been used as a marker for the selection of osteoblastic lineage cells from embryonic stem cells induced to differentiate into various lineages [55]. Runx2 is a transcription factor belonging to the Runx family, characterized by the highly conserved Runt domain. Runx2 is a specific transcription factor, and its expression is largely restricted to osteoblasts and mesenchymal condensations forming bones, cartilages and teeth. It plays a central role in osteoblast differentiation, chondrocyte maturation, bone formation and remodeling [56]. Runx2 heterozygous mutations have been identified in patients affected by Cleidocranial dysplasia, a dominantly inherited autosomal skeletal disorder characterized by open sutures and delayed closure of sutures, hypoplastic or aplastic clavicles, short stature, large fontanelles, dental anomalies and delayed skeletal development [57]. In this paper, a Tissue Microarray (TMA)-based immunohistochemical evaluation of the expression of β-catenin, cadherins (E-cadherin, N-cadherin, P-cadherin, OB-cadherin) and P-Runx2 was performed in formalin fixed, paraffin embedded samples from nine patients affected by fibrous dysplasia (n.8) and HPT-JT syndrome (n.1), and in seven controls, represented by reactive fibromyxoid tissue, reactive mature fibrous tissue, normal bone with hematopoietic cells and remodeling bone surrounding developmental odontogenic cyst.

According to our findings, β-catenin plays a central role in fibrous tissue proliferation and accompanies the lack of differentiation of osteoblast precursors in mature osteoblasts in FD. This study on the one hand confirms the complete absence of E-cadherin and OB-cadherin, but on the other hand shows that this protein loss is vicariate by the considerable increase in N-cadherin expression. Generally, N-cadherin over-expression has been detected in osteoblasts lining osteoid. Differently, in another case N-cadherin over-expression was observed in fibroblastoid cells committed to inefficient osteoblast differentiation. We observed hyperosteocytic bone, accommodating several osteocytes over-expressing N-cadherin, an aspect that could be interpreted as a consequence of abnormal cell matrix and cell–cell interactions [24].

P-cadherin showed a minor role, showing over-expression in a pagetoid/hyper-cellulated FD and a weak expression in a case of FD with the Chinese alphabet pattern. P-Runx2 showed over-expression in six out of eight cases of FD, both in fibroblastic stroma and in retracted osteoblast, and stained moderately positive in the rimming lining osteoblasts in HPT-JT syndrome. FD and HPT-JT were considered as syndromic models for the selective study of the altered process of differentiation, without increase or deregulation of the cellular cycle; they are in fact two pathological entities with low proliferative index, as valued by Ki67.

In our work, we also identified diverse histological characteristic patterns in FD and HPT-JT of bone, valuing the proportions of osteoid versus calcified bone, the fibrous cellularity, the deposition of collagen and the presence of fibroblast-like osteoblasts.

The limitations of the present work are attributable to the small size of the study sample and the retrospective nature of the study, for which it was not possible to prospectively assess the variation of the analyzed biomarkers in the context of disease progression and in relation to therapy with current medications available.

Surely, however, the evaluation of statistically significant differences in the expression of these biomarkers in correlation with histological patterns compared to the control group makes it possible to consider them as possible auxiliary factors in the complex framework of the diagnostic algorithm of fibro-osseous lesions.

## 5. Conclusions

We retain that the recognition of such patterns, the evaluation of the histological characteristics, the attribution of a corresponding score to any lesion, and the complete histochemical and immunohistochemical analysis of key molecules involved in osteoblast differentiation are essential for the diagnosis and classification as well as the appropriate clinical therapeutic management of fibrous-osseous lesions.

## Figures and Tables

**Table 1:** Clinical and histopathological findings in congenital fibro-osseous lesions of the jaw compared to reactive fibrous-osseous lesions and normal bone.

	Clinical Data							
Case N.	Age	Sex	Diagnosis	Anatomical Site	Bone/Osteoid Pattern: Pagetoid (P) Chinese Alphabet (CA) Hyper-Cellulated (HC)	^∇∇^ Cellularity 1. Low 2. Moderate 3. High	Hemorrhage 0. Negative 1. Positive	Rimming Osteoblasts 0. Negative 1. Focal 2. Moderate 3. High
1	49	M	FD	Maxilla	P/HC	3	0	2
2	44	M	FD	Maxilla	CA	2	0	1
3	37	M	FD	Mandible	P	1	1	1
4	31	M	FD	Mandible	P	1	1	1
5	25	F	FD	Mandible	CA	3	1	1
6	30	F	FD	Mandible	CA	3	0	1
7	35	F	FD	Maxilla	P	1	0	1
8			FD		CA	3		1
9	37	F	HPT-JT	Maxilla	P	1	0	1
Controls					Bone pattern			
10	26	M	Reactive fibromyxoid	Mandible	Parallel bone	2	0	1
11	60	M	Mature reactive fibrous tissue	Maxilla	Bone absent	1	0	0
12	23	M	Reactive fibrous tissue	Mandible	Parallel bone	2	0	1
13	27		Reactive fibromyxoid	Maxilla	Bone absent	2	0	
14	60	F	Mature reactive fibrous tissue	Maxilla	Bone absent	1	0	0
15	40	F	Normal bone with hematopoietic cells	Iliac crest	Trabecular bone	2	0	0
16	21	M	Remodeling bone surrounding developmental odontogenic cyst	Maxilla	Parallel trabeculae; focal fish-hook appearance	2	0	2

Legend: ∇∇, fibrous tissue findings; 1, low cellularity and collagen deposition; 2, moderate cellularity; 3, high cellularity.

**Table 2:** TMA based immunohistochemical findings in fibro-osseous lesions of the jaws.

	Immunohistochemical Findings									
Cases	Ki-67	CK AE1/AE3	Beta-Catenin			E-Cadherin	N-Cadherin	OB-Cadherin	P-Cadherin	P-Runx2
	Cell Count/HPF °	Score	Sub-Cellular Localization	Score	Nuclear Score	Score	Score	Score	Score	Score
1	6	0	C	3+Os	0	0	3+Os Fbs	0	3+	3+Os, Fbs
2	0	0	CM	3+Os	0	0	3+Os Octs	1+	0	3+Os, Fbs
3	4	0		0	0	0	3+	0	0	3+Os, Fbs
4	1	0	C	2+Os	0	0	2+Os; 0 Fbs	0	0	0
5	3	0	C	3+Os Fbs	0	0	2+Os Fbs	0	1+	3+Os, Fbs
6		0	-	0	0	0	1+	0	0	2+Os, Fbs
7		0	-	0	0	0	3+	0	0	0
8	5	0	CN	3+Os Fbs	3+	0	3+	0	0	3+Os, Fbs
9	0	1+	C	1+	0	0	1+	0	0	2+Os, Fbs
Controls								-		
10	4	0	M	0	0	0		0	0	1+
11	2	0	-	0	0	1+	0	0	0	1+
12	4	0	M	0	0	0		0	0	1+
13	5	0	M	1+	0	1+	NV	0	0	1+
14	6	0	-	0	0	0	2+Fbs	0	0	2+
15	0	0	M	1+	0	1+	1+	2+	1+	1+
16	4	0	M	1+	0	0	2+	3+	1+Os	2+

Legend: **CK**, cytokeratin AE1/AE3; **Ki-67**, proliferation index as evaluated by Mib-1 Ab. **M**, only membranous staining; **C**, only cytoplasmic; **C-M**, mixed membranous and cytoplasmic localization of beta-catenin, main cytoplasmic; **C-N**, mixed cytoplasmic and nuclear localization of beta-catenin, main cytoplasmic. **Os**, osteoblasts. **Octs,** osteocytes. **Fbs**, fibroblastoid appearing osteoblastic precursors. **NV**, non-valuable. **Score,** mean percentage of immune-labeled cells as evaluated by optical microscope and scored semi-quantitatively: 0, negative; 1+, <25; 2+, 25–50; 3+, 50–75; 4+, >75. ° Count of immunostained cells in 4 high power field (HPF).

## Data Availability

No new data were created or analyzed in this study. Data sharing is not applicable to this article.
